# Aquaporins and CO_2_ diffusion across biological membrane

**DOI:** 10.3389/fphys.2023.1205290

**Published:** 2023-06-13

**Authors:** Junyu Chen, Ke Yue, Lulu Shen, Chuncui Zheng, Yiyong Zhu, Kun Han, Lei Kai

**Affiliations:** ^1^ School of Life Sciences, Jiangsu Normal University, Xuzhou, China; ^2^ Hangzhou Institute of Test and Calibration for Quality and Technology Supervision, Hangzhou, China; ^3^ Jiangsu Provincial Key Laboratory for Organic Solid Waste Utilization, National Engineering Research Center for Organic-Based Fertilizers, Jiangsu Collaborative Innovation Center for Solid Organic Waste Resource Utilization, Nanjing Agricultural University, Nanjing, China; ^4^ Jiangsu Keybio Co., Ltd, Xuzhou, China

**Keywords:** carbon dioxide, aquaporin, biological membranes, diffusion, physiological relevance

## Abstract

Despite the physiological significance of effective CO_2_ diffusion across biological membranes, the underlying mechanism behind this process is not yet resolved. Particularly debatable is the existence of CO_2_-permeable aquaporins. The lipophilic characteristic of CO_2_ should, according to Overton’s rule, result in a rapid flux across lipid bilayers. However, experimental evidence of limited membrane permeability poses a challenge to this idea of free diffusion. In this review, we summarized recent progress with regard to CO_2_ diffusion, and discussed the physiological effects of altered aquaporin expression, the molecular mechanisms of CO_2_ transport via aquaporins, and the function of sterols and other membrane proteins in CO_2_ permeability. In addition, we highlight the existing limits in measuring CO_2_ permeability and end up with perspectives on resolving such argument either by determining the atomic resolution structure of CO_2_ permeable aquaporins or by developing new methods for measuring permeability.

## Introduction

More than 30 years ago, aquaporin was found to be a highly specialized water channel protein in erythrocytes (Preston et al., 1992; Agre, 2004). The discovery of aquaporins changed our perspective on the highly controlled permeability of biological membranes, which had previously been explained by the paradigm of free diffusion of water transport across membranes (Edidin, 2003). Aquaporins are a class of structurally conserved proteins that have been shown to function as channels for a wide range of neutral chemicals since their discovery. These molecules include glycerol (Jensen et al., 2001; Nollert et al., 2001), urea (Ishibashi et al., 1994; Ma et al., 1997), hydrogen peroxide (Almasalmeh et al., 2014), ammonia (Jahn et al., 2004; Kirscht et al., 2016), and even the gas molecule-carbon dioxide (Uehlein et al., 2003). Such a wide range of substrate selectivity suggests that biological membrane permeability is tightly controlled and is not just based on passive diffusion across the lipid bilayer. More than a century ago, Meyer and Overton proposed that the membrane permeability of a given solute is closely associated with its lipid solubility [also known as Overton’s rule (Missner and Pohl, 2009)]. For many molecules, experimental evidence confirmed this rule (Missner et al., 2008a), but some did not follow the prediction. The appearance of these molecules begs the question of whether or not Overton’s rule alone can account for the passage of molecules through biological membranes. Among those molecules that deviated from the prediction by Overton’s rule, CO_2_ was intensively investigated due to its role as a physiological component for essential process, i.e., respiration and photosynthesis. Despite the predicted high permeability of CO_2_, accumulated evidences from biologists found that certain cell membranes are remarkably resistant to CO_2_, which could not be explained solely by Overton’s rule due to its high lipophilic property. To address this challenge, Pohl’s team has introduced the effect of unstirred layers (USLs) and buffer, which account for a significant portion of the diffusion barrier of the lipid bilayer (Missner and Pohl, 2009). While research directed by Kaldenhoff and Boron independently demonstrated the existence of aquaporin-mediated CO_2_ transport in regulating the CO_2_ diffusion across biological membranes (Nakhoul et al., 1998; Uehlein et al., 2003). Since then, discussion has been continued with regard to the potential biological significance of CO_2_ channels in regulating CO_2_ transport across biological membranes. As more relevant results continue to uncover the complexities of CO_2_ movement across biological membranes, several questions have emerged: Why do some biological membranes have such low intrinsic CO_2_ permeability? How do biological systems deal with the conflict between the need for fast gas exchange and the low intrinsic permeability of the CO_2_ membrane? Is there a CO_2_ channel protein that exists in addition to free diffusion?

Baring the above open questions, we focused in this review on recent updates since our last systematic review article in 2014 (Kaldenhoff et al., 2014). We began with discussing the classical theory of CO_2_ solubility-diffusion of CO_2_ transport across lipid bilayers and the physiological influence of altered aquaporin expression. We then delved into the molecular details of aquaporin-mediated CO_2_ transport, as well as the role of sterols and nonrelevant membrane proteins on the overall CO_2_ permeability of biological membranes. Finally, we summarized the current limitations of the different methods used to measure CO_2_ permeability and offered a perspective on the current understanding of CO_2_ diffusion across biological membranes.

### Meyer overton’s rule and the CO_2_ solubility-diffusion model

As the basic principle for mass transportation through diffusive means, Adolf Fick described that the diffusive flux (*J*) was related to the diffusion coefficient (*D*) and gradient of the substrate (in case of two phases, rewritten as the concentration difference between the two phases ∇φ ) concentration:

J=−D∇φ (1)

Later, Meyer and Overton established the rule of spontaneous permeation of solutes and solvents across membranes, stating that the flux of the substance across a membrane, *J*, was linearly dependent on the permeability of the membrane, *P*_m_, with a concentration difference $∆c_{s}$ at two surfaces of the membrane, when the partition coefficient of the substance, *K*_p_, was given.

Eq. 1 can be rewritten as following:

$$J=-P_{m}∙∆c_{s}$$ (2)

Where $P_{m}=K_{p}∙D_{m}∕d$ , *D*_*m*_ is the diffusion coefficient, *d* is the thickness of the membrane.

As indicated by the rule, the permeability of a given molecule is related to the partition coefficient $K_{p}$ . Therefore, gas molecules, such as CO_2_ would have a membrane permeability as fast as permeating a water layer, with a $K_{p}$ ≥1. However, experimental data has shown contradictory results with extremely low gas permeability from certain biological membranes. As proposed by Pohl’s group, the existence of an unstirred layer, which dominates the resistance of CO_2_ diffusion, and the variation in the thickness of this layer could explain for this discrepancy (Missner et al., 2008b). In 2011, a joint correspondence letter was published that summarized the main agreement and disagreement on channel protein-mediated CO_2_ diffusion by the research groups Boron, Gros and Pohl (Boron et al., 2011). In summary, they agreed that channel-mediated CO_2_ transport would require a high resistance of the non-channel part of the membrane to CO_2_ diffusion and relatively low resistance to CO_2_ from USLs. In 2015, further cross-talk was initiated to collect new comments or views on CO_2_ transport mediated by channel proteins under physiological conditions by Cooper, Occhipinti, and Boron (Cooper et al., 2015). In this proposal, a new “access-solubility-diffusion-egress” model was proposed, where resistance of non-channel proteins, different headgroup of lipids, the role of cholesterol, as well as USLs, all accounted for the apparent CO_2_ permeability of biological membranes. While the disagreement still remains, Pohl pointed out the concern of data generated by both stopped flow and mass spectrometry, due to the fast process of CO_2_ diffusion in the range of milliseconds. Furthermore, it could be the limited availability of carbonic anhydrases (CAs), which led to extremely low CO_2_ permeability to the apical membranes. Finally, new points were raised: 1) How to explain the role of sterols and high percentage of membrane proteins, on the diffusion of diffusion of CO_2_ of biological membranes? 2) Mutation work that influences the function of certain aquaporin, resulting in the change of $P_{M,{CO}_{2}}$ , could not be correctly mapped to the change in thickness of USLs. 3) The altered activity CA activity of certain cells was not correlated with CO_2_ permeability. 4) The existence of USLs still challenged the proponents of CO_2_ channels.

### Physiological roles for aquaporin-mediated CO_2_ membrane diffusion

CO_2_ and O_2_ are gas molecules that play crucial roles in respiration by providing energy through oxidative phosphorylation reactions. Both gases need to be exchanged efficiently between the cellular organelles and the atmosphere, guided by their osmotic gradients. Unlike animals, plant cells or other photosynthesis microorganisms take up CO_2_ as a substrate for photosynthesis, and the concentration gradient is less significant compared to animals (Uehlein et al., 2017). Therefore, a higher efficient diffusion of CO_2_ from the atmosphere to the chloroplast stroma, where photosynthesis occurred, would be more beneficial for photosynthesis-active organisms (Kaldenhoff et al., 2014).

For quite a long time, the resistance of the mesophyll to CO_2_ was overlooked for green-leaf plants. Instead, the regulation of stroma and CO_2_ interconversion to bicarbonate and protons catalyzed by carbonic anhydrases (CA) was considered to be the limiting factor in CO_2_ availability (Kaldenhoff, 2012). However, even with complete deletion of CA activity in the chloroplast stroma, photosynthesis decreased by only about 7% (Price et al., 1994; Kaldenhoff, 2012). Furthermore, the mesophyll CO_2_ conductance varied rapidly in response to temperature, light, or water stress, instead of having a relatively constant value. This contradicted the pure physical model of mesophyll CO_2_ diffusion. Together, these shreds of evidence pointed to the existence of other major factors that regulated CO_2_ diffusion, such as aquaporin-mediated transportation.

The physiological influence of altered expression of potential permeable CO_2_ aquaporins was recently systematically evaluated and reviewed by Evans‘ group (Groszmann et al., 2017). To understand the role of certain putative permeable aquaporins with CO_2_, transgenic plants were generated and the impact on parameters relevant to photosynthesis was determined. In general, the change of mesophyII conductance was correlated with the tuned expression level of corresponding aquaporins. However, the mesophyII drawdown should be negatively correlated with the mesophyII conductance and the CO_2_ assimilation rate, where causal links between AQP and mesophyII conductance can be established. To provide the general ranges of photosynthetic-related parameters under varied mesophyII conductance, they performed simulations by changing the mesophyII conductance, when either stomatal conductance or *C*_i_ was set to be constant. They gave an estimated range of mesophyII drawdown, CO_*2*_ assimilation rates, transpiration rate, and transpiration efficiency based on consistent literature data (Groszmann et al., 2017). Furthermore, mesophyII conductance is a combined feature that could be influenced by many factors other than membrane permeability, such as the chloroplast surface area, adjacent to the intercellular air space per unit of leaf area and cell wall thickness (Evans, 2021). Since 2014, more direct or indirect evidence accumulated, supporting the aquaporin-facilitated CO_2_ transportation, i.e., AtPIP2;5, SlPIP1;2 (from tomato), OsPIP1;2 and OsPIP1;3 (from rice), HvPIP2;1, 2;2, 2;3, 2;5 (from Barley), ZmPIP1;5, 1;6 (from Maize), as well as SiPIP2;7 from C4 plant-foxtail millet (see Table 1).

**TABLE 1:** Summary of CO_2_ permeable aquaporins.

Names	Origins	Validation methods	References
NtAQP1	*Nicotiana tabacum*	*X. laevis* oocytes^a^, Yeast^b^, Black lipid membrane/copolymers^c^	Uehlein et al. (2003), Otto et al. (2010), Uehlein et al. (2012a), Kai and Kaldenhoff (2014)
AtPIP1;2	*Arabidopsis thaliana*	Leaf^d^, Yeast, *In vivo* ^e^	Heckwolf et al. (2011), Uehlein et al. (2012b)
HaPIP1;1	*Helianthemum almeriense*	Yeast	Navarro-Rodenas et al. (2013)
ZmPIP1;5	*Zea mays*	Yeast	Heinen et al. (2014)
ZmPIP1;6	*Zea mays*	Yeast	Heinen et al. (2014)
OsPIP1;2	*Oryza sativa L.*	*In vivo*	Xu et al. (2019)
OsPIP1;3	*Oryza sativa L.*	*In vivo*	Chen et al. (2021)
SlPIP1;2	*Solanum lycopersicum*	*In vivo*	Zhang et al. (2021)
NtPIP2;1	*Nicotiana tabacum*	Black lipid membrane	Uehlein et al. (2003), Uehlein et al. (2012a), Kai and Kaldenhoff (2014)
HvPIP2;1	*Hordeum vulgare L.*	*X. laevis* Oocytes	Mori et al. (2014)
HvPIP2;2			
HvPIP2;3			
HvPIP2;5			
AtPIP2;1	*Arabidopsis thaliana*	*X. laevis* oocytes	Wang et al. (2016)
AtPIP2;5	*Arabidopsis thaliana*	Yeast	Israel et al. (2021)
SiPIP2;7	*Setaria italica*	Yeast, *In vivo*	Ermakova et al. (2021)
PtAQP2	*Phaeodactylum tricornutum*	Mass spectrometry^f^	Matsui et al. (2018)
AQP1	*Homo sapiens*	*X. laevis* oocytes, Proteoliposome^g^	Nakhoul et al. (1998), Prasad et al. (1998), Musa-Aziz et al. (2009), Geyer et al. (2013)
AQP5	*Homo sapiens*	*X. laevis* oocytes	Wang and Boron (2019)
AQP5	*Rattus norvegicus*	*X. laevis* oocytes	Musa-Aziz et al. (2009), Geyer et al. (2013)
AQP6			
AQP9			
AQP0	*Bos taurus*	*X. laevis* oocytes	Geyer et al. (2013)
AQP1a1	*Danio rerio*	*In situ* ^h^	Talbot et al. (2015)
SsAqpZ	*Synechococcus* sp.	Yeast	Ding et al. (2013)

^a^ *X.laevis* oocytes: CO_2_ permeability was determined by a pH electrode that recorded the change in pH value when AQP was expressed in *X. laevis* oocytes (Geyer et al., 2013).

^b^ Yeast: CO_2_ permeability was determined by a stopped flow spectrophotometer when AQP was expressed in the yeast protoplast (Otto et al., 2010).

^c^ Black lipid membrane/copolymers: The permeability of CO_2_ permeability was determined by scanning the pH electrode when AQP was incorporated into a triblock copolymer or phospholipid bilayer (Uehlein et al., 2012a; Kai and Kaldenhoff, 2014).

^d^ Leaf: The same setup as the black lipid membrane except that a leaf patch instead of an artificial bilayer was measured (Uehlein et al., 2012b).

^e^ *in vivo*: the CO_2_ permeability was determined by the altered mesophyII conductance or photosynthesis related parameters via aquaporin overexpression or knockout mutant lines.

^f^ Mass spectrometry: The CO_2_ permeability was determined by following the O^18^ exchange monitored by mass spectrometry.

^g^ Proteoliposome: CO_2_ permeability was determined using a stopped flow spectrophotometer using aquaporin reconstituted liposomes.

^h^ *in situ*: aquaporin knockdown mutant zebrafish larvae were monitored by CO_2_ excretion using a custom-built total CO_2_ analyzer.

Recent studies have shown that the influence of altered expression of potential CO_2_ permeable aquaporins on the mesophyII conductance and photosynthesis rate should be calibrated by growth and environmental conditions, as well as the oligomeric/phosphorylation status of the corresponding aquaporins. Although there are accumulated evidences for aquaporin-mediated CO_2_ transportation, there have also been studies that have shown that simple manipulation of these aquaporins did not lead to changes in mesophyII conductance or photosynthetic efficiency. In one study, the knockout of three aquaporin genes-*AtPIP1;2*, *AtPIP1;3*, *AtPIP2;6* from *Arabidopsis thaliana* did not result in changes in mesophyII conductance nor photosynthetic efficiency. The authors discussed possible reasons for these results: i) functional redundancy within aquaporin families; ii) the possible change in hydraulic conductance together with the higher light intensities (200 μmol m^−2^ s^−1^) altered the photosynthetic capacity, which would be sufficient to remove the effect on both *g*_m_ and *g*_s_; iii) altered the hydraulic conductance of mutant lines through functional stimulation by colocalization of PIP1s and PIP2s on the plasma membrane (Kromdijk et al., 2020). However, the hydraulic conductance of mutant lines was not measured in the above study, which left this question to be further investigated. In another case, the ectopic expression of either *AtPIP1;*2 or *AtPIP1;4* in tobacco did not further increase mesophyII conductance nor the rate of assimilation of CO_2_. Similarly, the authors pointed out the influence of plant growth and environmental conditions on the ability of certain CO_2_ permeable aquaporins to alter mesophyII conductance, particularly, when a high basal *g*_m_ was observed in control wild-type control plants (Clarke et al., 2022). This effect was also observed from rice PIPs (Huang et al., 2021) and tomato SiPIP1;2 knockout mutants (Kelly et al., 2014), where *g*_m_ was affected only when grown in a CO_2_ enriched environment. Other studies have pointed out that the oligomeric or phosphorylation state of overexpressed CO_2_ permeable aquaporins can directly impact their function (Otto et al., 2010; Maurel et al., 2015; Groszmann et al., 2017). Additionally, aquaporins can act as signaling molecules, responding to different environmental stimuli and regulating stomatal dynamics in response to changes in ambient CO_2_ concentration (Ding and Chaumont, 2020) or ABA-mediated biotic stress (Fang et al., 2019). Finally, one important aspect to consider is the relative humidity within the substomatal cavity, which was assumed to be saturated when calculating the intercellular CO_2_ concentration determined by the gas exchange experiment (Cernusak et al., 2018). As recently investigated by Farquhar’s group, the relative humidity within the substomatal cavity could drop down to around 80%, with the saturation edge retreating to the mesophyII cell walls. Surprisingly, the mesophyII conductance to CO_2_ remained less affected when alter the Δ*w* (the difference between saturated humidity and the humidity in the air) if compared to uncorrected data, which might be controlled by the aquaporins within the mesophyII cell membranes (Wong et al., 2022). Although there are several aquaporins reported to function as both water and CO_2_ channels, the detailed mechanism of such potential dual functions still needs to be investigated, which could be investigated with new methods such as *in situ* measurement of water potentials within leaves using the fluorescent powder-hydrogel nanoreporters (Jain et al., 2021), as well as cell specific overexpress experiment to avoid functional redundancy from endogenous aquaporins using plant leave single cell RNA sequence data base (Kim et al., 2021).

To conclude, the impact of changes in CO_2_ permeable aquaporins on mesophyll conductance and photosynthesis rate should be considered, with respect to growth and environmental conditions, in particular the relative humidity within the substomatal cavity, as well as the oligomeric and phosphorylation status of the corresponding aquaporins.

### Molecular mechanism of aquaporin-mediated CO_2_ diffusion

Since the discovery of the CO_2_ channel protein: aquaporin-1 from humans and NtAQP1 from tobacco, many aquaporins from different organisms were reported to mediate CO_2_ transport, covering many members from mammals, plants, microalgae, and fish (see Table 1). The family of aquaporins has a relatively conserved structure, with six membrane-spanning helices, two reentrant short helices with NPA motifs, and flexible N-/C-termini heading towards the cytosol. The six bundle-like membrane-spanning alpha helices were tightly arranged in a circle, constituting the solute conduction pore/channel. Although aquaporins function as the water channel in monomers, they often form a quaternary tetramer assembly in native membranes and even large orthogonal arrays in the case of AQP4 (Ho et al., 2009). Until now, the physiological relevance of such a tetrameric assembly is not completely clear; however, a few cases showed that the central pore formed by the aquaporin tetramer was likely to be the CO_2_ channel. Early work based on *X. laevi*s oocytes with low intrinsic CO_2_ permeabilities provided experimental evidence that AQP1 acts as a permeable CO_2_ channel (Nakhoul et al., 1998). Later, a molecular simulation based on the high-resolution structure of AQP1 gave the atomic level of details that the central pore of the AQP1 tetramer could mediate fast CO_2_ diffusion in low intrinsic CO_2_-permeable membranes (Hub and de Groot, 2006). This hypothesis was further demonstrated by the yeast protoplast system to determine the altered permeability of CO_2_ when the assembly of the artificial tetramer with a fixed ratio of NtPIP1;2 and NtPIP2;1, connected by a short linker (Otto et al., 2010). The results demonstrated that the homo-tetrameric assembly of CO_2_ permeable NtPIP1;2 was necessary for CO_2_ channel activity. However, such a relationship between the oligomeric state and CO_2_ permeability was not further investigated in other model plants, except for tobacco.

**FIGURE 1:**
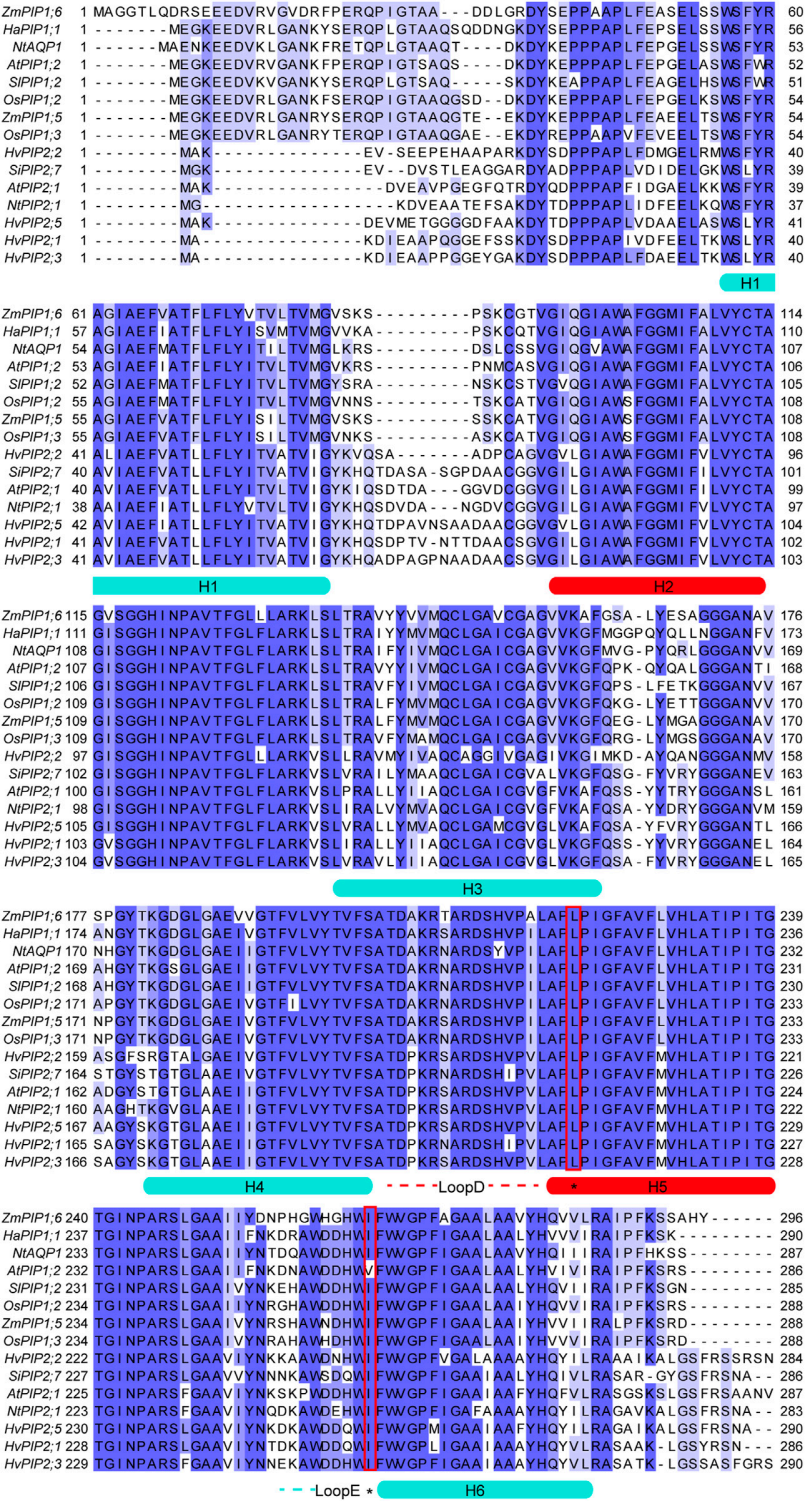
Sequence alignment of CO_2_ permeable aquaporins from plants. Alignment was performed by the online tool Claustral Omega (https://www.ebi.ac.uk/Tools/msa/clustalo/) using default settings (Madeira et al., 2022). Blue shades indicated the percentage of identity. Helical regions were highlighted and denoted H1-H6. The central pore lining region were highlighted in red, including H2, H5 and LoopD. The key residues for CO_2_ permeability were highlighted with red rectangles.

In 2021, Tyerman et al. gave a systematic review on multifunctional aquaporins, describing the dynamic regulation of the central pore with the high-resolution crystal structures of AQP1 and SoPIP2;1 (Tyerman et al., 2021). Furthermore, the MOLEonline MOLEonline channel radii analysis (Berka et al., 2012) showed a diameter of 3.6 Å at the Leu200 constriction residue, modelled by the closed water channel conformation of SoPIP2;1 (PDB: 1Z98) (Tornroth-Horsefield et al., 2006). According to the analysis, the constriction side would allow the CO_2_ to pass when considering a kinetic diameter of 3.3 Å for CO_2_. Additionally, Tyerman et al. (2021) proposed that both post-translational modification and protein-protein interactions could contribute to dynamic regulation of central pore permeability via local conformational changes, allowing a wide range of molecules, including K^+^, Na^+^, as well as CO_2_ passing through the central pore. In early studies, aquaporins from the PIP1 family were found to be permeable to CO_2_. While, later on, members from the PIP2 family were also reported to function as CO_2_ channels. Despite the relative conservation of transmembrane helices in all PIP aquaporins, it was difficult to identify the crucial residues creating the selective filter of the central pore, which was not surprising given the very variable pore environment generated by tetrameric assembly. As seen in Figure1, the major part of the central pore lining area is composed of transmembrane helices 2 and 5 and loop D, which were dynamically influenced by other neighboring motifs as well. However, due to the lack of high-resolution structure of PIP1 aquaporin, it remains unresolved how the sequence difference between PIP1s and PIP2s could contribute to CO_2_ permeability, especially the long N-terminal flexible loop that only exists in PIP1s. Although it is difficult to obtain structural details for the flexible loop region, a detailed biochemical assay might answer this question, such as domain switch or truncated variants in the case of an N-terminal loop. As indicated in Figure 1., conserved residues Leu in helix 5 and Ile at the end of loop E were reported to be essential to allow the passage of a CO_2_ molecule based on either simulation or biochemical assays (Mori et al., 2014; Tyerman et al., 2021). However, other residues alone the channel might also be the restriction site, depending on the arrangement of the helixes structures that form the central channel.

### Role of sterols and non-CO_2_ permeable proteins and technical challenges in measuring CO_2_ permeability

The Singer-Nicolson fluid-mosaic model was widely recognized as the fundamental model for the structure and molecular dynamics of the plasma membrane (Singer and Nicolson, 1972). Many basic properties of biological membranes were characterized on the basis of this two-dimensional fluid model. Among the basic properties, the permeability was also intensively investigated using such a lipid bilayer model both theoretically and experimentally. However, other factors, such as sterols or integrated membrane proteins were not considered, which could influence the overall permeability (Suzuki and Kusumi, 2023). Therefore, lack of such factors could be the potential source for the inconsistency of measured membrane permeability. This inconsistency became non-trivial when determining CO_2_ permeability. Due to the higher lipophilic properties of CO_2_, the phospholipid-formed lipid bilayer exhibits very low resistance to CO_2_, while the plasma membrane of *X. laevis* oocytes, Madin-Darby canine kidney (MDCK) cells, the transformed human embryonal kidney SV40 cell line (tsA201), as well as the apical membrane of the gastric glands, showed extremely low CO_2_ permeability (Endeward et al., 2006a; Endeward et al., 2006b; Endeward et al., 2008; Itel et al., 2012). In a recent review, Gros et al. proposed that the cholesterol content in the majority biological membranes dominates its CO_2_ permeability, regulating the CO_2_ permeability by at least 2 orders of magnitude with a cholesterol content between 0%–70% (Arias-Hidalgo et al., 2018). However, an exception of normal native human red cells showed aquaporin-dependent CO_2_ permeability instead of cholesterol content, indicating the existence of unidentified factors (Endeward et al., 2008). Kaldenhoff’s group suggested a possibility, pointing out the role of non-channel proteins on the CO_2_ permeability of the phospholipid bilayer (Kai and Kaldenhoff, 2014). Finally, the existence of lipid rafts, which are rich in both sterols and proteins, could further contribute to the overall permeability [see review by Kai Simons and Elina Ikonen (Simons and Ikonen, 1997)].

One possible reason for the inconsistent permeability of CO_2_ reported in many previous reports could be the limitations of different techniques in determining permeability of CO_2_, due to the high permeability of the phospholipid bilayer (Endeward et al., 2014). Both stop flow-based and mass spectrometry-based methods were questioned for their inability to quantify dynamic fast CO_2_ across the membrane (Boron et al., 2011; Hannesschlaeger et al., 2019). On the other hand, the scanning pH electrode could provide an alternative that was not limited by the fast dynamics of CO_2_ passing through the black lipid membrane. However, the formation of a black lipid membrane with the solvent-containing method was challenged by the presence of organic solvent n-decan, as well as whether aquaporins still survive as a functional form during the formation of the corresponding black lipid membrane (Hannesschlaeger et al., 2019). Therefore, new techniques that can determine the fast transportation of CO_2_ across the membrane and avoid the influence of solvents may be necessary to improve the accuracy of the CO_2_ permeability measurements.

## Conclusion

Despite the numerous structural and functional studies of aquaporins in the past several decades, our understanding of the detailed mechanism of functional and structural diversity of these relatively conserved channel proteins is still in its infancy. The debate over whether aquaporins are permeable to CO_2_ continues, with accumulating both supportive and contradictory evidence. However, the challenges in directly measuring CO_2_ permeability across native or artificial membranes make it difficult to fully interpret the results and understand their physiological implications. More attention should be paid to the interpretation of the data and investigating the potential effects of aquaporin overexpression on plant cultivars and photosynthesis-related parameters. Ultimately, a technical breakthrough for the direct measurement of CO_2_ transportation through aquaporins would be needed to fully clarify the molecular details and bring an end to the ongoing debate.
